# Plasma channel formation in NIR laser-irradiated carrier gas from an aerosol nanoparticle injector

**DOI:** 10.1038/s41598-019-45120-3

**Published:** 2019-06-20

**Authors:** Eva Klimešová, Olena Kulyk, Yanjun Gu, Laura Dittrich, Georg Korn, Janos Hajdu, Maria Krikunova, Jakob Andreasson

**Affiliations:** 1 ELI Beamlines, Institute of Physics AS CR, v.v.i., Na Slovance 2, 182 21 Prague 8, Czech Republic; 2 Technische Universität Berlin, Institut für Optik und Atomare Physik, ER 1-1, Strasse des 17. Juni 135, 10623 Berlin, Germany; 3 Department of Cell and Molecular Biology, Uppsala University, Husargatan 3 (Box 596), SE-751 24 Uppsala, Sweden; 4 Chalmers University of Technology, Department of Physics, Göteborg, Sweden

**Keywords:** Atomic and molecular interactions with photons, Laser-produced plasmas, Characterization and analytical techniques

## Abstract

Aerosol nanoparticle injectors are fundamentally important for experiments where container-free sample handling is needed to study isolated nanoparticles. The injector consists of a nebuliser, a differential pumping unit, and an aerodynamic lens to create and deliver a focused particle beam to the interaction point inside a vacuum chamber. The tightest focus of the particle beam is close to the injector tip. The density of the focusing carrier gas is high at this point. We show here how this gas interacts with a near infrared laser pulse (800 nm wavelength, 120 fs pulse duration) at intensities approaching 10^16^ Wcm^−2^. We observe acceleration of gas ions to kinetic energies of 100s eV and study their energies as a function of the carrier gas density. Our results indicate that field ionisation by the intense near-infrared laser pulse opens up a plasma channel behind the laser pulse. The observations can be understood in terms of a Coulomb explosion of the created underdense plasma channel. The results can be used to estimate gas background in experiments with the injector and they open up opportunities for a new class of studies on electron and ion dynamics in nanoparticles surrounded by a low-density gas.

## Introduction

The development of laser light sources with high intensity ultrashort pulses has opened up new possibilities in science, from the study of structure and dynamics in structural and molecular biology^1–7^ to detailed studies of ionization/excitation processes in atoms/molecules^8–13^, to investigations of matter under extreme conditions^14–17^. In all of these research areas, methods of well-controlled sample delivery are of key importance.

New and very interesting research fields have been opened up by the start of operations of X-ray free-electron lasers. At such facilities aerosol injectors based on an aerodynamic lens stack (ALS)^18–22^, delivering a focused beam of nanoparticles, biomolecules, viruses or cells into a vacuum chamber, have been largely used for single-particle coherent diffractive imaging (CDI)^2–6,23–25^ and have been recently suggested also for serial femtosecond crystallography (SFX)^26^. Additionally, combined CDI and ion spectroscopy has brought new insights into understanding the explosion dynamics of individual single clusters and single particles mainly by eliminating the averaging effects^15–17,27–29^. Besides CDI and SFX applications, aerosol injectors are used for a number of investigations in atomic, molecular and optical (AMO) sciences, eg. to study ultrafast electron dynamics in single dielectric nanoparticles irradiated by NIR pulses^30–33^, or to map laser absorption on nanoscale by imaging ions after sample explosion^34^. A very promising field is nanoplasmonics, where irradiating individual (metal) nanoparticles with tuned laser pulses can result in significant electron acceleration by local inhomogenous fields on the nanoscale^35–37^. Using aerosol sample delivery methods together with electron and ion spectroscopy can widen the research area of nanoplasmonics by allowing new ways to separate sample and substrate effects.

In an aerosol injector the sample in a volatile buffer is first nebulized, often using either a gas dynamic virtual nozzle (GDVN)^38^ or an electrospray unit^23,39^, and then focused to a vacuum chamber via an ALS^18–22^. In this process a carrier gas is used: typically helium in GDVN, and a mixture of CO_2_ and nitrogen in electrospray units. The buffer solvent evaporates along the way to the exit of the ALS leaving single intact nanoparticles surrounded by the carrier gas. To achieve a high hit rate in a laser-nanoparticle experiment, a tightly focused particle beam is required. In practice aerosol beam diameters of <10 *μ*m have been reported^22,26^. This tight particle focus is produced at a short distance (few mm) from the ALS tip^22,26^ introducing spatial constraints for bringing any device between the ALS exit and the interaction region. When the nanoparticles are irradiated with an intense laser, not only signal from the sample is detected, but also carrier gas can contribute to ion, electron and/or photon emission resulting from the interaction. Several groups have employed differential pumping for electron and ion detection experiments to decrease the carrier gas density^30,31,34,40^, but in these cases the diameter of the particle beam was relatively large ∼0.5 mm. An alternative approach is to perform experiments with the regular injector on nanoparticles tightly focused by the high gas flow. This opens new possibilities to explore the complex interaction between an inhomogeneous medium and an intense laser field and can find applications eg. in nanoplasmonics where the local field enhancement in the vicinity of nanostructures can be exploited for enhanced light emission or electron acceleration^36,41–44^. Therefore, it is important to investigate the interaction of the carrier gas with intense laser fields to assess its influence on the complete sample dynamics^45^.

Here we present an extensive study on ion emission from gases (argon or helium) injected to the vacuum chamber via an aerosol injector developed at the Laboratory of Molecular Biophysics at Uppsala University (the “Uppsala injector”)^22^. This injector is based on a GDVN combined with ALS and will be used at the ELI Beamlines international facility in the Czech Republic at a user end-station for AMO sciences and CDI. The gas stream from the injector was hit by the femtosecond pulse-train of a NIR laser at power densities approaching 10^16^ Wcm^−2^. At these intensities, field ionisation dominates the interaction with NIR pulses. We note, however, that field ionisation does not take place with X-ray pulses at similar power densities. We show that after irradiation with a strong NIR laser pulse a plasma channel is formed in the interaction region where ions (coming both from the injector carrier gas and from the background gas in the chamber) are accelerated by Coulomb forces. Our experimentally found ion energies agree well with our calculations of ion Coulomb explosion in a plasma channel. Ions from the gas irradiated by a strong NIR pulse can be accelerated to energies of 100 s eV and thus, can significantly influence the ion and electron emission from the sample. Furthermore, we show how the measured maximum ion energies from the injector carrier gas can be used to estimate the gas density in the interaction region in future experiments with this type of sample delivery systems.

## Results

### Ion time-of-flight spectroscopy

In the experiment carrier gas from the injector was irradiated with a NIR laser (800 nm central wavelength, 120 fs FWHM pulse duration) focused to a peak intensity of 9 × 10^15^ Wcm^−2^. Ions originating from the interaction were detected by a time-of-flight (ToF) spectrometer in the direction perpendicular to both the laser and the injector axes with the ion spectrometer axis oriented parallel to the NIR beam polarization (see Methods).

Two parameters of the injector operation were varied: (i) the density of the gas flow inside the ALS and (ii) the distance between the ALS exit and the interaction point. Both parameters determine the density of the particles in the interaction region and thus the hit rate, and define the carrier gas density in the interaction region. For the first scan (i) we have chosen the readout *p* of the pressure gauge at the ALS entrance (see Methods for exact definition) as we expect this to be linearly related to the gas density in the interaction region. In the second scan (ii) the injector was moved away from the laser beam from its minimal experimentally-possible position of 11 mm above the laser focus in steps of ∼1.6 mm upwards to the top position of 46.4 mm from the interaction region.

#### Ion acceleration as a function of the gas density entering the ALS

**Figure 1:**
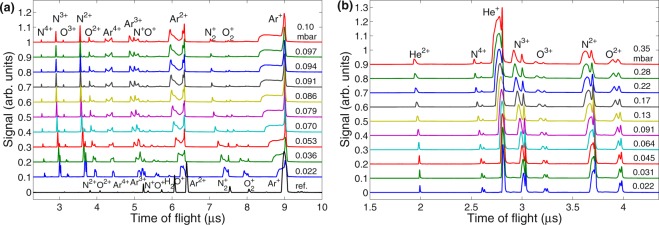
Ion time-of-flight (ToF) traces (vertically offset for clarity) of NIR laser-irradiated gas injected through the injector at different entrance pressures of the aerodynamic lens stack (ALS). Laser wavelength: 800 nm, pulse duration: 120 fs, peak intensity: 9 × 10^15^ Wcm^−2^, distance from the ALS tip: 11 mm. (**a**) Argon. Bottom line (black) shows a reference trace when chamber was back-filled with argon (injector not used). (**b**) Helium. ALS entrance pressure in mbar is shown next to each ToF trace.

Figure 1 shows charge-state resolved ion ToF traces applying argon (a) or helium (b) as a carrier gas through the injector. Note that with respect to the argon reference ToF trace (black line in Fig. 1(a)), which shows sharp atomic or molecular lines, all peaks in ToF traces measured with the injector are broadened towards shorter ToFs. In particular, with the increase of the ALS entrance pressure Ar^+^ peak develops an accelerated front that extends towards shorter ToFs (ie. higher kinetic energies). Similarly, Ar^2+^ signal is broadened and develops a peak at shorter ToFs and argon higher charge state peaks split into two. Analogously, He^+^ signal is broadened towards higher energies and He^2+^ ions get accelerated, when helium ALS entrance pressure rises (Fig. 1(b)). Comparably to carrier gases, ion signal from background gases (${{\rm{O}}\,}_{2}^{+}$, ${{\rm{N}}\,}_{2}^{+}$, O^+^, N^+^, O^2+^, N^2+^, O^3+^, N^3+^, N^4+^) develops a double-peak structure with one peak getting faster with increasing the ALS entrance pressure. Hence, all ions, coming both from the carrier and the background gas, gain energy in the interaction.

**Figure 2:**
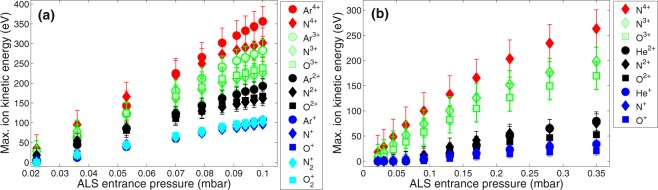
Maximum kinetic energies of carrier as well as background gas ions as a function of the entrance pressure of the carrier gas in the aerodynamic lens stack (ALS). Increasing the entrance pressure increases gas density at the interaction point located 11 mm from the ALS tip. Data are extracted from ion time-of-flight traces with (**a**) argon or (**b**) helium as a carrier gas. Groups of ions with the same charge state are plotted with the same colour.

To evaluate the ion acceleration process, we analyze maximum ion kinetic energies of N, O and Ar or He ions as a function of the ALS entrance pressure of the carrier gas (Fig. 2) assuming that ions with the shortest ToF have initial velocity in the direction towards the detector (see Methods). Both for argon and helium the ion energies rise with increasing the ALS entrance pressure. This is expected as higher ALS entrance pressure corresponds to higher gas density in the interaction region resulting in larger charge density to accelerate ions. Note that groups of ions with the same charge state are marked by the same colour in Fig. 2 to indicate that the kinetic energy gain is sensitive primarily to the ion charge state and not to the ion mass. For example, singly-charged ions ${{\rm{O}}\,}_{2}^{+}$, ${{\rm{N}}\,}_{2}^{+}$, O^+^, N^+^ and Ar^+^ have very similar energy for each ALS entrance pressure (Fig. 2(a), cyan and blue symbols).

#### Ion acceleration as a function of the distance from the injector tip to the interaction region

**Figure 3:**
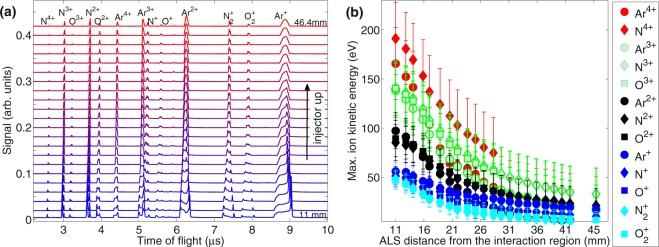
(**a**) Ion time-of-flight traces of NIR laser-irradiated argon for different distances *d* between the aerodynamic lens stack (ALS) and the interaction region. Laser wavelength 800 nm, pulse duration 120 fs, peak intensity 9 × 10^15^ Wcm^−2^, ALS entrance pressure 0.057 mbar. Traces are offset for clarity. (**b**) Maximum ion energy of argon and background ions from (**a**) as a function of the distance *d*. Groups of ions with the same charge state are marked by the same colour.

Figure 3(a) shows ion ToF traces as a function of the distance between the ALS tip and the interaction region for argon injection to the chamber with ALS entrance pressure of 0.057 mbar. The results of this scan can be scaled for higher pressures needed for nanoparticle focusing. Maximum ion energies extracted from the experimental traces are shown in Fig. 3(b). As in Fig. 2, groups of ions with the same charge state are marked by the same colour. As it follows from Fig. 3, ion acceleration is most prominent at a short distance from the ALS tip. After moving the injector aproximatly 30 mm away, ion energies are approaching zero within the experimental error, and the ToF traces start to resemble traces with low-density gas. When the injector is close to the laser focal spot (and thus particles are well focused, if injected) the acceleration of the carrier and the background gas ions is substantial.

Results from the ALS pressure scan (i) and the injector distance scan (ii) show that the ion kinetic energy gained in the interaction with the strong laser field does not depend on the mass but only on the ion charge and the charge density. We attribute these findings to the Coulomb explosion mechanism. In the following section we present a theoretical model showing that the electrons, which are field-ionised by the strong NIR laser field, are driven out of the focus by the laser ponderomotive forces and a channel of underdense plasma is formed behind the pulse. As a result the unscreened positively charged sample undergoes a Coulomb explosion.

### Calculation of the Coulomb explosion of the plasma channel

We calculate the explosion of the plasma channel in two steps: (i) We perform particle-in-cell (PIC) simulations of electrons driven by the laser field to show the channel formation and obtain parameters of the channel. Ion motion is not included in this calculation because ions are too slow to move on the time scale considered. (ii) We analytically calculate the potential energy of an ion at a surface of a charged cylinder using values from the first step. We assume that the potential energy is converted to the kinetic energy of the ion. We then compare the measured ion kinetic energies with the calculated ones.

#### Numerical simulation of the channel formation

The simulation starts with a pre-ionised underdense plasma. In the numerical calculation a laser propagates in the *x* direction through the underdense plasma and the motion of electrons driven by the laser ponderomotive force is calculated (for details see Methods). The calculation was performed for a laser with 800 nm wavelength, 120 fs pulse duration and peak intensity of 10^16^ W cm^−2^. To obtain the electron dynamics for a range of densities that can be present in the experiment the gas density was scanned in the range of 10^13^–10^17^ cm^−3^.

**Figure 4:**
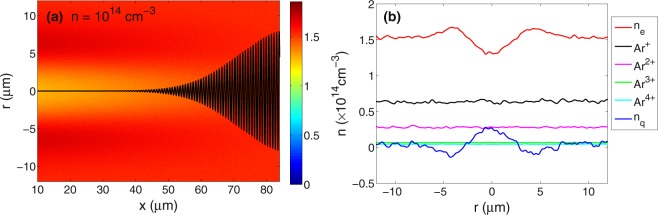
(**a**) Calculated electron density distribution at time 540 fs for laser intensity 10^16^ Wcm^−2^, wavelength 800 nm and argon density *n* = 10^14^ cm^−3^. The black line shows the laser electric field. (**b**) The density profiles of electrons, different ion charge states and the total charge *n*_*q*_ along a chosen line *x* = 16 *μ*m for the same parameters as in (**a**).

To illustrate the channel formation, we show in Fig. 4 the electron density distribution and individual density profiles calculated for argon gas density of 10^14^ cm^−3^ at time 540 fs (for definition of time zero, see Methods). A plasma channel is formed behind the pulse, where a Coulomb explosion with associated ion acceleration is expected from the uncompensated positive charge. The positive background left after the pulse is not balanced by fast electron motion, therefore, the channel is still present after the laser pulse.

This type of behaviour is observed over the range of gas densities studied here. Going to higher densities, the charge separation becomes less pronounced, because there is a higher charge holding electrons in the focal volume. For gas densities higher than ~5 × 10^16^ cm^−3^ the charge separation becomes negligible and other mechanisms (mainly pressure of hot electrons) are expected to drive the ion explosion.

From this numerical calculation we extract certain parameters of the plasma channel: the electron perturbation rate is *θ* = *δn*_*e*_/*n*_*e*_ ≈ 0.15 (for definition see Methods) and the channel radius roughly equals the focal spot radius *R* ≈ 5 *μ*m. These parameters do not change considerably in the density range 10^13^–10^15^ cm^−3^ and are used for the analytical calculations describing the ion explosion.

#### Analytical calculation of maximum ion energies

To calculate the ion kinetic energy from the computed charge profiles we approximate the plasma channel by a thin cylinder with length *L* and linear charge density *l*_*q*_ = *n*_*q*_*πR*^2^, where *n*_*q*_ = *θ*〈*Z*〉*ne* is the volume charge density inside the cylinder, 〈*Z*〉 is the average charge inside the cylinder, *n* is the gas density and *e* is the electron charge. The length of the cylinder is taken to be the Rayleigh length (*L* ≈ 100 *μ*m for our case).

We consider an ion with charge *Z* at distance *R* from the cylinder axis. The ion is placed at the ion spectrometer axis. The potential energy of the ion is:

1 $$E=\frac{\theta Z\langle Z\rangle ne{R}^{2}}{4{\varepsilon }_{0}}\,\mathrm{ln}[\frac{\sqrt{{L}^{2}+4{R}^{2}}+L}{\sqrt{{L}^{2}+4{R}^{2}}-L}],$$

where *ε*_0_ is the vacuum permittivity. For the energy calculation the electron perturbation rate is taken to be *θ* ≈ 0.15 based on the PIC simulations and 〈*Z*〉 is calculated by tunnel ionisation rates^46^ at the end of the laser pulse. We assume that the potential energy from Equation (1) is converted to the ion maximum kinetic energy and we compare it with the experimental values in the next section.

### Comparison of experimental and calculated ion energies

**Figure 5:**
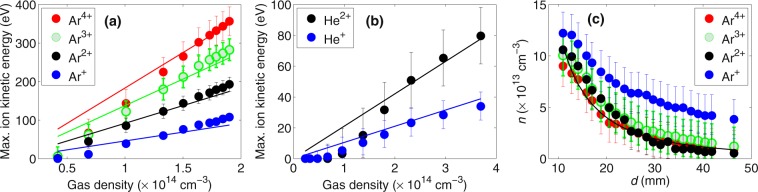
(**a**,**b**) Symbols – measured maximum ion energies (same data as in Fig. 2), lines – calculated ion energies, (**a**) argon, (**b**) helium. (**c**) Argon gas density calculated from the measured ion energies from the injector position scan (Fig. 3(b)). Legend indicates from which ion charge state the density was calculated. Solid line shows theoretical curve.

To compare calculated maximum ion energies with experimental values from the ALS pressure scan we assume a linear scaling between the gas density in the interaction region and the ALS entrance pressure. We introduce a scaling constant *C* to obtain the gas density (see Methods). Fig. 5(a,b) show the measured (symbols) and calculated (lines) energies of argon and helium ions as a function of the gas density in the interaction region. Measured ion energies are the same data as in Fig. 2 and the horizontal axis was converted from the ALS pressure to the gas density by choosing the scaling constant *C* = 0.044. There is a good agreement between measured and calculated ion energies for a large range of gas densities.

To compare experimental data from the injector position scan with the calculated values, we first calculate gas density as a function of distance *d* from the ALS tip using Equation (1) for all charge states of argon, see Fig. 5(c). With the exception of Ar^+^, all charge states result in the same density dependence on *d*, indicating consistency of the model. A small discrepancy of densities obtained from Ar^+^ ions may be due to the uncertainty of the extraction of the shortest ToF from the broad Ar^+^ signal in the ToF trace. Also the final distribution of Ar^+^ might be influenced by the charge recombination.

To further support our model, we compare measured densities with a theoretical curve of gas flow from the injector. We assume density scaling from the injector tip^47,48^ as *n*(*d*) = *n*_0_/(1 + *Bd*)^2^, where *B* is a parameter directly related to parameter *C* above (see Methods). The density dependence on *d* calculated in this way is shown as a black line in Fig. 5(c) and is in a reasonable agreement with densities obtained from the measurement. Thus, our model captures well the main physical processes underlying the interaction of the injected gas with a strong NIR laser field and can be used for estimation of gas density in future experiments. We note that there are uncertainties and assumptions in our approach, but there is no need to build an additional experimental setup (eg. an interferometer) to measure gas density. So when one performs experiments on injected nanoparticles interacting with strong NIR laser field, the gas background can be estimated by an ion measurement without nanoparticle injection.

## Discussion

We have performed an extensive study on ion emission from NIR laser-irradiated gases injected into a vacuum chamber through a GDVN combined with an ALS. In our measurements with argon or helium as carrier gases, we observe ions accelerated to energies of 100s eV. For a given charge state and experimental conditions (ALS entrance pressure or type of carrier gas) the ion energy depends only weakly on the ion species, indicating a Coulomb explosion process. We support our experimental findings by a calculation of a Coulomb explosion of a plasma channel created by the NIR laser pulse in a gas medium. We characterize the gas ionization as a function of the ALS entrance pressure and of the distance between the ALS tip and the interaction region and use this to estimate the carrier gas density at the interaction point. Our findings are important to consider when using this type of sample delivery system for electron and ion spectroscopy with NIR intense laser fields and could find use as a non-invasive on-line diagnostics tool of the carrier gas density in future experiments with the injector, including single particle CDI.

When designing an experiment on ion and/or electron emission from injected nanoparticles one has to take into account the presence of the carrier gas. For certain applications differential pumping can be implemented between the ALS tip and the interaction region to decrease the carrier gas density. Another possibility is to work without the differential pumping and close to the interaction region, where the particle density, and thus the hit rate, is higher, but where the carrier gas can significantly contribute to the sample dynamics. Experiments in this regime, with a controllable gas density surrounding isolated nanoparticles, bring new opportunities to the investigations of intense NIR laser interaction with gas-nanoparticle ensembles. It can be very promising e.g. for nanoplasmonics where local field enhancement on the nanoscale can be exploited to enhance electron and light emission from gases in the vicinity of nanostructures.

## Methods

### Laser system

Experiments were performed with an amplified Ti-Sapphire laser system (wavelength 800 nm) with a FWHM pulse duration of 120 fs at the target position and energy per pulse of 0.78 mJ. The beam was focused to a spot with 1/*e*^2^ diameter of 10 *μ*m, yielding a peak intensity of 9 × 10^15^ Wcm^−2^. The laser polarization was horizontal, parallel to the axis of the ion ToF spectrometer.

### Injector setup

Gas was introduced into a vacuum chamber using the “Uppsala injector”^22^. Argon or helium were brought into a vacuum chamber through the outer orifice of a manually fabricated GDVN^38,49^ with input pressures in the range from 20 to 200 psi (1.4 bar to 13.8 bar). The inner capillary dedicated for liquid sample was not used. After the nebulization chamber the gas propagated through a nozzle/skimmer stage for excess gas removal^50,51^ with nozzle diameter of 0.3 mm and skimmer diameter of 0.5 mm. After passing through the skimmer the gas entered the aerodynamic lens stack at a pressure between 0.022 to 0.35 mbar (ALS entrance pressure *p*) and was then injected to the chamber. We used these rather low ALS entrance pressures to avoid saturation of the ion peaks and observe contributions also from the background gas. The ALS entrance pressure was measured with a Pirani vacuum gauge (Edwards APG100) and the reading was corrected for different types of gas used. Pressure inside the interaction chamber was around 1 × 10^−8^ mbar without gas injection and raised up to 5 × 10^−6^ mbar when gas was injected.

The ALS tip was placed ∼11 mm from the interaction region, which was the minimum possible distance in our geometrical conditions. For the position scan the injector was moved away from the interaction region along the injector axis in steps of ∼1.6 mm upwards to the top position of 46.4 mm.

For a reference measurement without the injector (black bottom line in Fig. 1(a)) argon was introduced to the chamber through a 40 *μ*m diameter capillary. The input pressure to the capillary was 3.2 bar, which resulted in pressure in the chamber of 2.5 × 10^−7^ mbar.

### Ion ToF spectrometer

Ions created during the interaction of the laser pulse with the gas were detected with an ion ToF spectrometer in the direction perpendicular to both the laser and the injector axes. The ToF spectrometer consisted of a repeller and an extractor plate followed by a field-free region and then a microchannel plate detector. Repeller and extractor plates had dimensions of 50 mm × 20 mm and the distance between them was 10 mm. The interaction region was in the middle between the plates (within ∼100 *μ*m uncertainty from the exact central position, determined by fitting experimental ion peak positions with calculated values). For data presented here, 2000 V were applied on the repeller plate and the extractor plate was grounded. Both plates had a central hole with diameter of 2 mm to preferably select ions with the initial momentum close to the detector axis. With these spectrometer settings, ions with energies up to 400 eV with initial velocity in the opposite direction to the detector will turn around within the spectrometer plates and fly towards the detector. More energetic ions flying in the opposite direction can escape from the plates and, thus, will not reach the detector.

The maximum ion kinetic energies were extracted from the measured peaks in ion ToF traces in the following way. We calculated the ToF for a given ion with different initial energies. We assumed initial ion velocity to be towards the detector as this will result in the shortest ToF. Then we took the initial ion energy that matched the experimental position of the accelerated peak. The uncertainty of this calculation comes mainly from the ambiguity of the ToF axis calibration. The calculated ToF of an ion with a given initial energy largely depends on the exact position of the interaction region with respect to the repeller and extractor plates. Error bars in Figs 2, 3(b) and 5 were determined by calculating the ion initial kinetic energy for different ion’s initial positions, varied in the range ±50 *μ*m.

### Theoretical model of the plasma channel

The interaction of electrons and the laser field was simulated by the 2-dimensional particle-in-cell (PIC) simulations with the relativistic electromagnetic code EPOCH^52,53^. PIC simulation is a widely used and reliable method to investigate the dynamics in plasmas. In this method, physical particles are represented by a number of pseudoparticles. The fields generated by the laser pulse and the motion of particles are calculated by a Finite Difference Time Domain (FDTD) method. All the electromagnetic field components are calculated within a grid with fixed spatial resolution. The forces generated by these fields are applied on the pseudoparticles and used to update their velocities and positions. At the end of the loop, the new calculated pseudoparticles’ positions and velocities are used to update the fields again.

In the simulations, a linearly polarized Gaussian pulse with a peak intensity of 1 × 10^16^ Wcm^−2^ propagates along the *x*-axis in a gas medium. The laser axis is along *r* = 0 (*r* is the distance from the laser axis) and the simulation box starts at *x* = 0. The FWHM pulse duration is *τ* = 120 fs, laser wavelength *λ* = 800 nm and the spot size diameter (1/*e*^2^) is 10 *μ*m. The start of the simulation (time zero) is when the maximum of the Gaussian laser pulse is at position 2*τ* before the simulation box (at *x* = 0). The initial gas density was scanned in the range of 10^13^–10^17^ cm^−3^. The ionization process is not included in the calculation, initial ion charge states and electron density are taken from field ionisation calculation^46^ at the rising edge of the pulse. However, we note that the main outcomes of our analysis do not depend on the exact choice of initial charge state values. Due to the short time scale, the ion motion is negligible in the simulation. The mesh size in the simulations is *δx* = *δr* = 0.01*λ* and the simulation box has a size of 12000 × 7000 cells in longitudinal (*x*) and transverse (*r*) direction, respectively. About 1.44 billion of pseudoparticles are employed in the simulation. The time step is about 0.007*T*_0_, where *T*_0_ = 2.7 fs is the laser period. After the simulation the electron perturbation rate *θ* is calculated as *θ* = (*n*_*e*_(0) − *n*_*ef*_)/*n*_*e*_(0), where *n*_*e*_(0) is the electron density at time zero, ie. at the beginning of the calculation, and *n*_*ef*_ is the electron density on the laser axis (*r* = 0) at the end of the calculation.

### Comparison of theoretical and experimental ion energies

To compare calculated maximum ion energies for the ALS pressure scan with experiment we proceeded in this way. First, we calculated gas density corresponding to the ALS entrance pressure *p* from ideal gas equation: *n*_0_ = *p*/*k*_*B*_*T* (eg. gas density corresponding to *p* = 0.04 mbar is 10^15^ cm^−3^). We assume the gas density *n*(0) at the ALS tip (distance from interaction to the tip *d* = 0) equals the density *n*_0_ corresponding to the measured ALS entrance pressure. The gas density in the interaction region at distance *d* = 11 mm is assumed to be *n* = *Cn*_0_, where *C* < 1 is a scaling constant obtained by matching the calculated and experimental data for argon and helium pressure scans.

For the calculation of the gas density flowing from the ALS tip, we use the scaling *n*(*d*) = *n*_0_/(1 + *Bd*)^2^. Parameter *B* is related to *C* by known densities at two points along the gas streamline: *n*(0) = *n*_0_ and *n*(11 mm) = *Cn*_0_, (giving $B=(\sqrt{1/C}-1)/11\,$mm). This procedure allows to use one parameter *C* to fit three experimental scans (argon pressure scan, helium pressure scan and argon position scan).

To easily estimate the gas density in future injection experiments without performing the PIC simulations we suggest the following procedure:

1. Measure *E*(*p*) – ion kinetic energies *E* as a function of the ALS entrance pressure *p*, and measure *E*(*d*) – ion kinetic energies *E* as a function of the distance *d* between the interaction region and the ALS tip. It is beneficial to perform measurements with different gases to obtain more experimental values for comparison with the calculation.
2. Calculate ion kinetic energies as a function of the gas density using Equation (1). We note there are some uncertainties in the variables entering Equation (1), namely in the average charge state 〈*Z*〉, assumed symmetry and charge distribution in the channel and the electron perturbation rate. For a practical comparison, one can introduce an additional parameter *A* to include these uncertainties. Accordingly, one gets for the pressure scan:
  2 $$E(p)=\frac{AC\theta Z\langle Z\rangle e{R}^{2}p}{4{\varepsilon }_{0}{k}_{B}T}\,\mathrm{ln}[\frac{\sqrt{{L}^{2}+4{R}^{2}}+L}{\sqrt{{L}^{2}+4{R}^{2}}-L}],$$
  and for the injector position scan:
  3 $$E(d)=\frac{A\theta Z\langle Z\rangle e{R}^{2}p}{4{\varepsilon }_{0}{k}_{B}T{(1+(\sqrt{1/C}-1)d/{d}_{0})}^{2}}\,\mathrm{ln}[\frac{\sqrt{{L}^{2}+4{R}^{2}}+L}{\sqrt{{L}^{2}+4{R}^{2}}-L}],$$
  where *d*_0_ is the distance between the interaction region and the ALS tip at which the pressure scan was performed.
3. Find parameters *A* and *C* to get best fit with the experimental data. Note that the nonlinear dependence of ion energy on *C* in Equation (3) makes it possible to find reliable values of the two parameters *A* and *C*.

## Data Availability

The datasets generated during the current study are available from the corresponding author on reasonable request.
